# Super-enhancers: a new frontier for epigenetic modifiers in cancer chemoresistance

**DOI:** 10.1186/s13046-021-01974-y

**Published:** 2021-05-19

**Authors:** Guo-Hua Li, Qiang Qu, Ting-Ting Qi, Xin-Qi Teng, Hai-Hong Zhu, Jiao-Jiao Wang, Qiong Lu, Jian Qu

**Affiliations:** 1 Department of Pharmacy, the Second Xiangya Hospital, Central South University; Institute of Clinical Pharmacy, Central South University, 139 Middle Renmin Road, Changsha, Hunan 410011 People’s Republic of China; 2 Department of Pharmacy, Xiangya Hospital, Central South University, Changsha, 410008 People’s Republic of China

**Keywords:** Super-enhancer, Chemoresistance, Epigenetic reprogramming, Cancer, Therapy

## Abstract

Although new developments of surgery, chemotherapy, radiotherapy, and immunotherapy treatments for cancer have improved patient survival, the emergence of chemoresistance in cancer has significant impacts on treatment effects. The development of chemoresistance involves several polygenic, progressive mechanisms at the molecular and cellular levels, as well as both genetic and epigenetic heterogeneities. Chemotherapeutics induce epigenetic reprogramming in cancer cells, converting a transient transcriptional state into a stably resistant one. Super-enhancers (SEs) are central to the maintenance of identity of cancer cells and promote SE-driven-oncogenic transcriptions to which cancer cells become highly addicted. This dependence on SE-driven transcription to maintain chemoresistance offers an Achilles’ heel for chemoresistance. Indeed, the inhibition of SE components dampens oncogenic transcription and inhibits tumor growth to ultimately achieve combined sensitization and reverse the effects of drug resistance. No reviews have been published on SE-related mechanisms in the cancer chemoresistance. In this review, we investigated the structure, function, and regulation of chemoresistance-related SEs and their contributions to the chemotherapy via regulation of the formation of cancer stem cells, cellular plasticity, the microenvironment, genes associated with chemoresistance, noncoding RNAs, and tumor immunity. The discovery of these mechanisms may aid in the development of new drugs to improve the sensitivity and specificity of cancer cells to chemotherapy drugs.

## Background

Cancer is presently a leading cause of death in 91 countries [1]. According to a report by the International Agency for Research on Cancer, there were 19.3 million new cases and nearly 10.0 million deaths from cancer in 2020 worldwide, and the incidence will increase in the future [2, 3]. Beyond traditional chemotherapy approaches, new therapies, including targeted therapies and immunotherapy, have attracted scientific attention and produced clinical applications [4, 5], however, tumor heterogeneity and resistance remain major obstacles to cancer treatment [6]. The resistance of tumor cells to chemotherapeutics (chemoresistance) is a critical challenge that oncological studies seek to understand and overcome [7].

Chemoresistance describes the reduced toxicity of chemotherapy drugs to tumor cells, which often leads to treatment failure. The responsiveness of tumor tissue to chemotherapy is determined by three main factors: the type of drug, the biological characteristics of the cancer cells, and the specific tumor microenvironment (TME) [8]. Most studies have focused on the internal factors of cancer cells, including cancer stem cells (CSCs), multi-drug resistant proteins (MDRPs), autophagy, DNA damage repair, and epigenetic regulation [9, 10]. Addressing each decisive factor separately can help solve the problem of chemoresistance.

Epigenetic regulation is a way of regulating cell phenotype without changing DNA sequence. Recent studies suggested that chemoresistance is involved in both genetic and epigenetic heterogeneities and highlighted the role of epigenetic regulations [11–17]. Chemotherapeutics induce epigenetic reprogramming in cancer cells, converting a transient transcriptional state into a stably resistant one [18, 19].

Super-enhancers (SEs), first discovered by Young *et al*. in 2013, are a large cluster of multiple enhancers that can greatly promote gene expression [20]. Although the total number of genetic control elements can reach into the millions, only a few hundred SEs control the key genes for cell identity and function [21]. SEs are important elements in epigenetic regulation and play a key role in the occurrence and progression of diseases, particularly cancer, and they have the potential to be developed into new therapeutic targets and diagnostic markers [22, 23]. There have been multiple studies on the mechanisms by which SEs affect chemoresistance, providing a new direction to overcoming obstacles in chemotherapy, but there have been few summaries in this field. Thus, we focus here on the emerging role of epigenetics, particularly SEs, on chemoresistance through regulation of the formation of CSCs, cellular plasticity, the microenvironment, the genes associated with chemoresistance, and non-coding RNAs (ncRNAs), to contribute new ideas to improve the efficacy of chemotherapy.

## Chemoresistance overview

### Intrinsic and acquired chemoresistance

Chemoresistance may appear early in the process of tumorigenesis, whether de novo/primary (intrinsic) resistance or acquired/secondary resistance [24]. In intrinsic resistance, naive tumors do not produce a response to first-line chemotherapy in the initial treatment, whereas in acquired resistance, tumors are initially sensitive to chemotherapy, but it later fails to elicit a response [25]. Intrinsic resistance is selective, and it is related to genetic instability and tissue-specific chemoresistance-related gene expression [26]. Acquired resistance results from drug induction, meaning that the drug triggers transcription and signaling pathways related to apoptosis and anti-apoptosis [27, 28]. Some studies have shown that chemoresistance is the result of random mutations but is nevertheless drug specific [29–31]. There are also some similarities in gene regulation between intrinsic resistance and acquired resistance, including autophagy, mutation of target proteins, and the overexpression of MDRPs [32, 33]. Both intrinsic and acquired resistance may exhibit multidrug and cross-resistance to agents that are structurally and pharmacologically diverse [34]. Tumor heterogeneity in resistance development has attracted more interest lately. Tumor heterogeneity is at the foundation of intrinsic and acquired chemoresistance, which can refer to patient heterogeneity, inter-tumor and intratumor cellular heterogeneity, genomic heterogeneity including mutations and gene fusion, and epigenetic heterogeneity with inherent differences between cell populations, as well as the possibility of therapy-induced epigenetic changes [35]. Due to the heterogeneity of tumors on the molecular and cellular levels, many mechanisms can coexist within tumors to induce chemotherapeutic resistance [35, 36].

### Mechanisms of chemoresistance

**Fig. 1:**
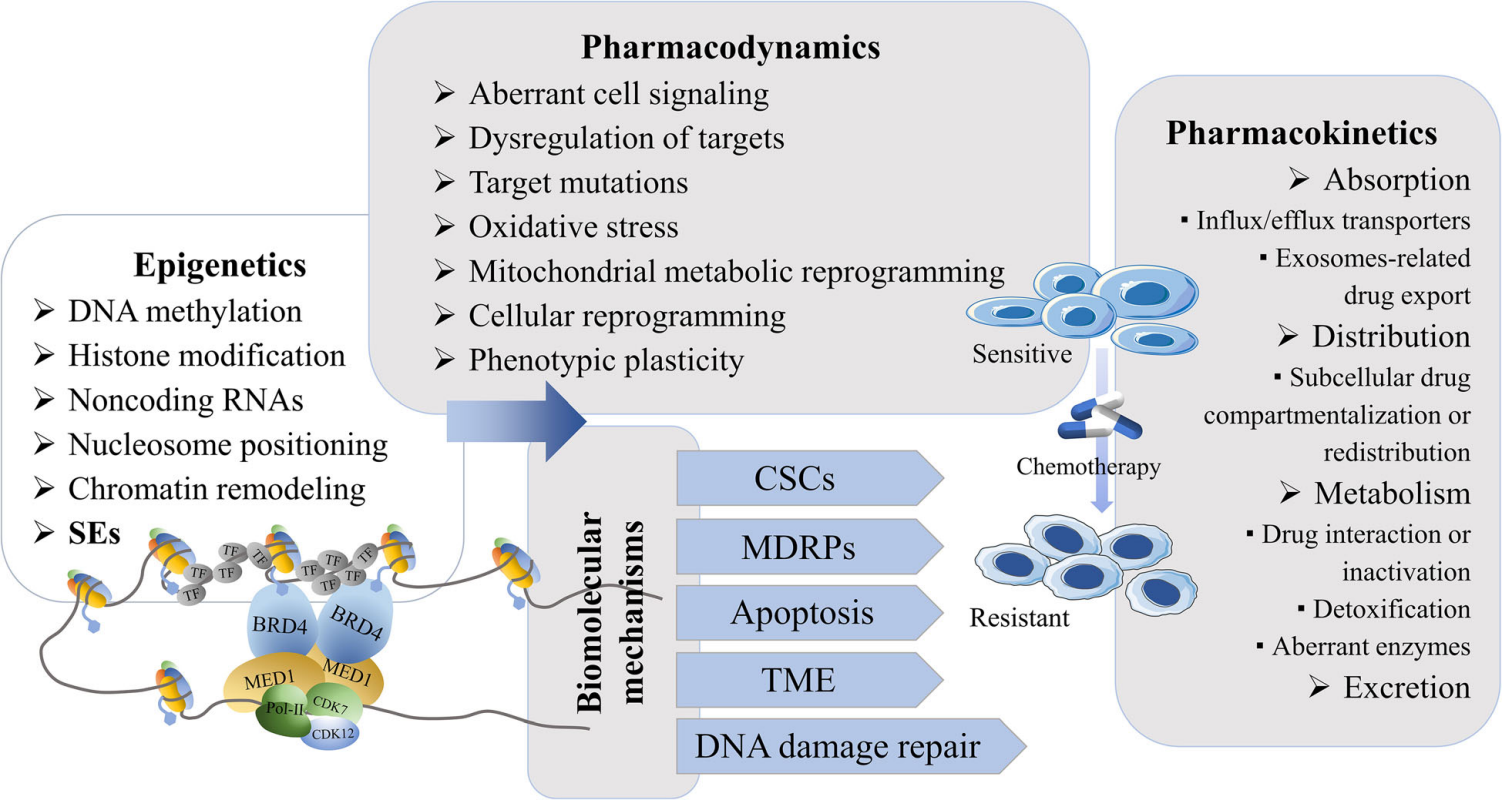
Pharmacokinetic and pharmacodynamic factors leading to tumor chemoresistance and related mechanisms. Various factors, including many biomolecular mechanisms, are involved in the induction of chemoresistance through influencing the pharmacokinetics and pharmacodynamics factors of chemotherapy drugs. Epigenetic regulation, particularly through SEs, plays an important role in this process

The development of chemoresistance involves several mechanisms at the molecular and cellular levels [11]. The complex mechanisms that cross-talk and interact with each other in chemoresistance are founded in the pharmacokinetics and pharmacodynamics of chemotherapy drugs [11] (Fig. 1). The factors that affect the pharmacokinetics (absorption, distribution, metabolism, and excretion) of chemotherapeutic drugs include drug transporter-mediated change in drug influx/efflux, exosome-mediated drug export, subcellular drug compartmentalization and redistribution, and altered drug metabolism (which involves changes in drug interaction, inactivation, detoxification, and aberrant drug-metabolizing enzymes) [37, 38]. The mechanisms of pharmacodynamic chemoresistance include aberrant cell signaling, dysregulation in target expression and function, high-frequency mutations that target enzymes or change their catalytic function, genetic instability, oxidative stress, mitochondrial metabolic reprogramming, changes to the microenvironment, cellular reprogramming and phenotypic plasticity, inefficient apoptosis, and DNA repair [11, 37, 38]. Multiple biomolecular mechanisms are involved in the development of chemoresistance in cancer cells, including, but not limited to, CSCs, overexpression of MDRPs (e.g., P-glycoprotein; P-gp), dysregulation of apoptosis, TME, DNA damage repair, and epigenetic dysregulation [9].

Epigenetics refers to genetic changes in cell phenotypes that have nothing to do with changes in DNA sequences; the word is often used to describe the regulation of chromatin during DNA replication, transcription, and repair [39]. Related mechanisms include DNA methylation, histone modification, ncRNAs, and nucleosome positioning [40, 41]. DNA methylation leads to tighter chromatin, which inhibits gene expression. Conversely, acetylation modification increases chromatin accessibility and changes the nucleosome positioning to promote gene expression [42].

There is growing evidence that chemoresistance is not only related to genetic changes but is also influenced by epigenetic regulation. Epigenetics has shed light on the elaborative cellular machinery involved in both tumor development and chemoresistance [11, 43]. The epigenetic landscape of cancer cells includes both heterogeneity and plasticity, as well as associated alterations [44]. Chemosensitive tumors that respond to primary chemotherapy can relapse but still respond to second-line chemotherapy, in a pattern that is attributable to heterogenicity and the relatively stable epigenetic state, while chemoresistant clones within a chemosensitive tumor may accrue temporal epigenetic changes during chemotherapy that then would change to a stable chemoresistant epigenetic state [43, 44]. Chemotherapeutics induce epigenetic reprogramming in cancer cells, converting a transient transcriptional state to a stably resistant one [19, 45]. Further, genetic changes, such as mutations in the regions of epigenetic regulating factors, can induce epigenetic aberrations, including changes in DNA methylation, histone covalent modifications, nucleosome repositioning, and SE landscape changes [46].

ncRNA refers to RNA that does not encode a protein, including ribosomal RNA, transfer RNA (tRNA), small nuclear RNA, small nucleolar RNA, microRNA, long non-coding RNA (lncRNA), circular RNA, and ncRNAs with unknown functions [47]. ncRNAs play a vital role in gene regulation, either by participating in base complementary pairing, or by acting as scaffolds or molecular chaperones for chromatin regulation [48]. Enhancer RNAs (eRNAs) and SE RNAs (seRNAs), transcribed by enhancers or SEs, in turn regulate the activity of enhancers or SEs through a variety of mechanisms, such as interacting with RNA polymerase II (RNA pol-II), promoting histone acetylation, and increasing transcription factor (TF) recruitment and chromatin accessibility [49–51].

The mechanism of epigenetics in tumor tolerance has been confirmed by multiple studies. DNA methylation downregulates the expression of antigen processing and presentation molecules, such as MHC I and Fas, leading to immune escape and reducing the sensitivity of the tumor cells to immunotherapy [52]. Histone demethylation can alter the chromatin state so that the cells dynamically survive drug exposure, that is, a single cell is in a transient and reversible tolerant state [53]. Similarly, epigenetic modifications also occur in CSCs, where DNA methylation and histone modifications regulate the activity of key signaling pathways, including wnt/β-catenin, Hedgehog, and Notch, and the expression of ATP-binding cassette transporter proteins [54]. Moreover, ncRNAs also play an important role in the chemoresistance of various cancers, such as hepatocellular carcinoma (HCC) [55], colorectal cancer [56, 57], gastric cancer [58], lung cancer [59], and pancreatic cancer [60].

Many reviews have described the role that epigenetics plays in chemoresistance [12, 43, 44], but there is still insufficient detail on the function of SEs in chemoresistance. In the following sections, we describe the general components of SEs, followed by a detailed discussion of the potential association between SE aberrations and the mechanisms of chemoresistance.

## Structures and functions of SEs

### Concept and structures of SEs

The enhancer is a non-coding cis-regulatory element, bounded by TFs, cofactors, mediators, and RNA Pol-II, that is responsible for transcription regulation in the human genome [61]. SEs are a large cluster of enhancers with a length of 8–20 kb that are enriched in more TFs, cofactors, mediators, RNA Pol-II, and histone H3 lysine27 acetylation (H3K27ac) than typical enhancers [21]. Cyclin-dependent kinase 7 (CDK7) and bromodomain-containing protein 4 (BRD4) are also important components of SEs and are enriched in SE regions [62]. High signals of H3K27ac and histone H3 lysine4 methylation (H3K4me1) usually represent active enhancers, and H3K27ac ChIP-seq is widely used to identify SEs [63].

**Fig. 2:**
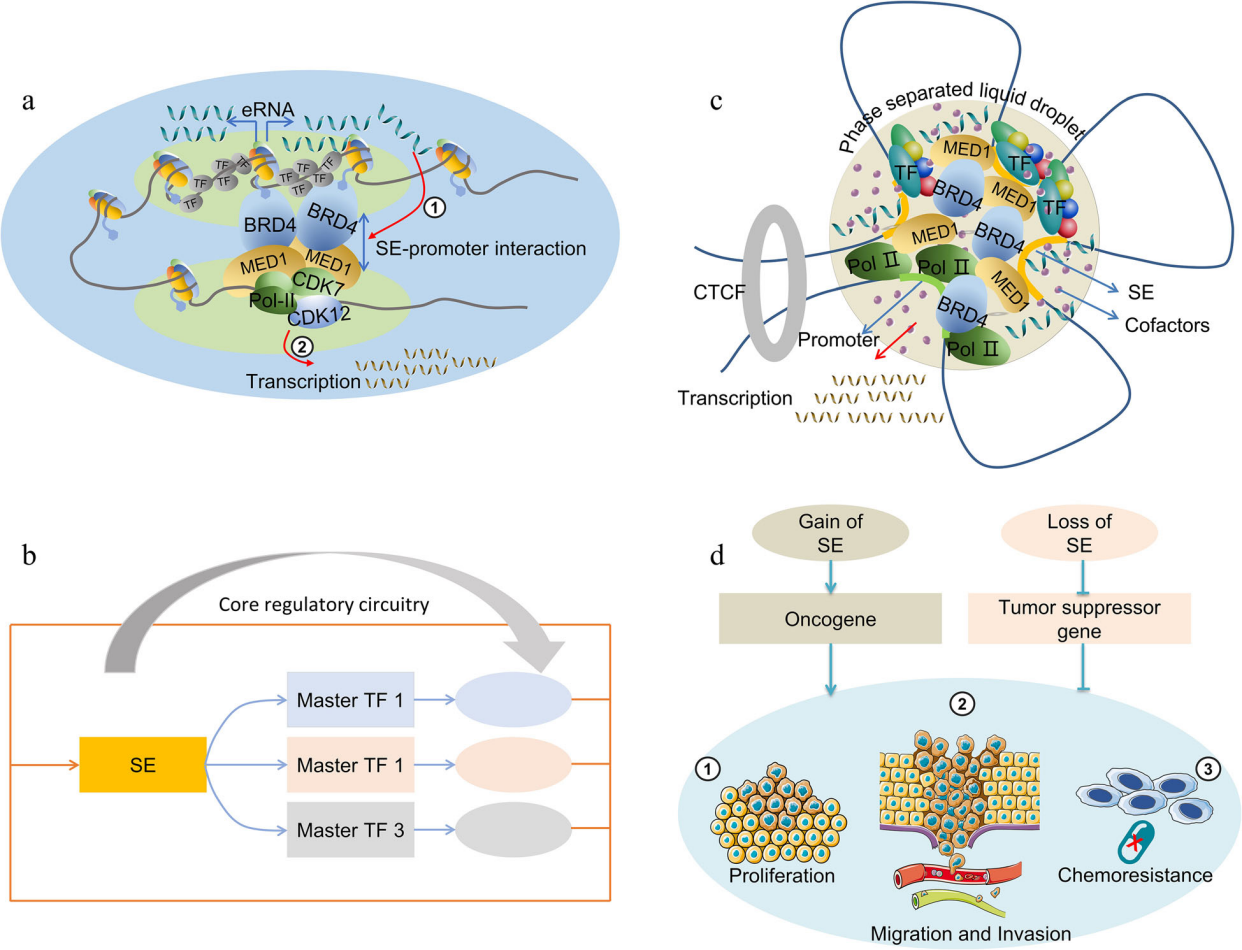
Structure and function of SEs. **a** eRNAs transcribed from SE regions enhance the SE-promoter interaction and contribute to the transcription of target genes. **b** Master TFs form a core transcriptional regulatory circuitry by binding to their SE regions and strongly promote their own expression. **c** Multiple components including TFs, cofactors, MED1, BRD4, and RNA Pol II form a phase separation structure in the SE region, which promotes cross-link interactions and concentrates the transcription apparatus at SE-associated genes. **d** Gain or loss of SEs increases tumor proliferation, invasion, and chemoresistance through promotion of the expression of oncogenes or inhibiting the expression of tumor suppressor genes

SEs strongly upregulate the expression of target genes by forming a physically interacting SE-promoter loop consisted of SEs, target genes, TFs, cofactors, mediators and RNA Pol-II, which spatially narrows the distance between SE and the promoter through cohesion [64]. Interestingly, the target gene is usually located either downstream or upstream of the SE, indicating that the regulation of SE is directionless [65]. Moreover, the distance between the SE and its target gene is uncertain, and SE usually acts through distant chromatin interactions [66]. Therefore, it may be that SEs simultaneously regulate the expression of multiple genes and may not follow rules of proximity [67]. ncRNAs transcribed from the SE region mediated by RNA Pol-II are called eRNAs [68]. Studies have shown that these eRNAs promote the formation of the SE-promoter loop and contribute to SE activity [69, 70] (Fig. 2a). Various TFs may occupy the SE region, among which some core TFs could regulate their own expression through SE-promoter interaction, thus forming a core transcriptional regulatory circuitry (CRC) [71, 72]. The CRC model can help us to better understand the role of SE in cell type-specific transcriptional regulation (Fig. 2b).

The three-dimensional (3D) conformation of chromatin influences gene expression and biological processes since DNA is packed in chromatin [73]. Studies have shown that the 3D organization of chromatin is dynamic in the regulation process of gene expression, and 3C (chromosome conformation capture) and its extended technologies including 4C (circularized chromosome conformation capture), 5C (chromosome conformation capture carbon copy) and Hi-C (high-throughput chromosome conformation capture) are often used for conformation research of chromatin [74].

SEs are usually located in the SE domains (SDs), specifically within the topologically associating domain (TAD) [75]. TADs, regions enriched in chromatin interactions, are composed of contact domains and multiple subTADs containing dense genes and inhibitory and activating chromatin signatures [76]. TADs are chromatin loop architectures formed in the process of genome organization, and are basic units of 3D nuclear organization, the properties and functions of which are affected by the 3D conformation of chromatin [77–79]. Architectural proteins, architectural protein binding sites, tRNAs, short interspersed nuclear elements and housekeeping genes form the boundaries of TADs that play a role of insulator and guarantee the interactions of distant elements [77, 80]. CCCTC-binding factor (CTCF) is an important architectural protein that can associate with proteins such as transcription factor IIIC, condensins and cohesins to build a TAD boundary at a specific genomic location, thereby preventing cross-site interactions [81]. Insulated by the strong boundaries with lower chromatin interaction frequencies, SEs can only target genes within the SDs, thus preventing abnormal SE-promoter interactions and transcriptional activation [80, 82]. In addition, mediated by low-complexity disordered regions or intrinsically disordered regions, SEs can form membraneless phase-separated structures, which concentrate biologically and physically similar proteins or other molecules, thus enabling efficient transcription [83]. The transcriptional coactivators BRD4 and mediator of RNA Pol-II transcription subunit 1 (MED1) were found to form condensates at SEs, thereby compartmentalizing and concentrating the transcription apparatus [84]. Moreover, eRNAs serve as a scaffold for SE phase separation [69]. Hnisz *et al*. established a phase separation model to explain the transcriptional control of SEs, which is helpful for us to understand the formation and function of SEs as well [85] (Fig. 2c).

### Roles of SEs in cancer

#### SEs control cell identity

The factors that induce the formation of oncogenic SEs include DNA mutations and indels, chromatin rearrangements, changes in the 3D structure of the chromosome, and viral infections [86]. In the unique SE-promoter 3D loop and phase separation structure, SEs usually show greater transcriptional activation ability than typical enhancers [21]. SEs are also more sensitive to perturbations and thus can be targeted by small molecular inhibitors such as JQ1, a BRD4 inhibitor, and THZ1, a CDK7 inhibitor [87]. Previous studies have shown that SEs regulate the expression of cell-type-specific genes [88, 89]. Therefore, SEs can be considered powerful cis-regulatory elements, defining cell identity and conferring cell fate.

It has been reported that SEs contribute to the maintenance of stem cell identity, including ESCs [83], hair-follicle stem cells [90], and hematopoietic stem cells [91]. In addition, SEs can regulate uterine development [63], T cell development [92], and myogenic differentiation [93]. However, the recurrent gain or loss of SEs usually leads to diseases, including neurodegenerative disease [94], autoimmune disease [75], and various cancers [20]. SEs undergo dynamic remodeling in the progression of cell lineage [95]. The formation of SEs for key TFs associated with the control of cell identities, such as Oct4, Sox2 and Nanog, can reprogram cell fate through CRCs in ESCs, even in cancer cells [21, 96]. Research by Denes Hnisz *et al.* showed that SEs have the potential to become biomarkers of specific cancers, which may provide references for the occurrence, diagnosis and treatment of cancers [20].

#### SEs drive tumorigenesis, tumor progression, and prognosis

Aberrant gene transcription driven by SEs can always lead to tumorigenesis, tumor progression, and prognosis [97]. Therefore, SEs can be used as effective biomarkers for cancer diagnosis, treatment, and prognosis. Moreover, the identification of cancer-specific SEs can help us to find new critical oncogenes and uncover novel mechanisms for different cancers. On this occasion, many SE-related databases, including dbSUPER [98], SEA version 3.0 [99], and SEdb [100], have been established to facilitate SE exploration. Due to the development of genome-wide epigenetic data, tumor epigenetic markers such as SEs have been attracting more and more attention for their use in predicting tumor progression, prognosis/disease free survival, chemo sensitivity/chemoresistance, and recurrence.

In HCC, an important regulatory axis related to SE was found: transcription factor 4 (*TCF4*) occupies the SE region and induces extensive interactions between SE and the AJUBA promoter, which strongly promotes AJUBA expression and increases cancer metastasis [101]. In addition to directly regulating the expression of coding genes directly, SEs can also regulate the expression of ncRNAs in cancer [102]. seRNAs are transcribed from the SE and can recruit TFs, promote the formation of SE-promoter loops, direct chromatin accessibility, and regulate SE acetyltransferase activity [51]. Jiao *et al*. found that heparinase eRNA enhances chromatin looping between the SE and promoter in several cancer cell lines, promoting tumorigenesis *in vitro* and *in vivo* [103]. *Klf6* is a gene responsible for tumorigenesis, and the loss of the *Klf6* SE was found to inhibit the proliferation of liver cancer cells by upregulating *miR-1301* [104]. lncRNA *HCCL5* is an SE-driven gene that confers the malignant phenotypes of liver cancer cells [105]. LINC00162, an SE lncRNA, was highly expressed in bladder cancer cells and tissues, which can promote progression of bladder cancer [106]. Our team constructed prognostic models for five -genes associated with SEs for osteosarcoma patients and multiple myeloma patients, which accurately predict the prognosis of these cancer patients [107, 108].

Studies that use the CRC model and are focused on master TFs and SEs are gradually growing in number. Zhang was recently the first to study SE-associated CRC transcriptional control in lung adenocarcinoma: master TFs *ELF3*, *EHF*, and *TGIF1* were found to co-occupy the SE region and promote each other’s expression through the formation of CRC, which induces the malignant progression of lung adenocarcinoma [109]. Similarly, in Ewing sarcoma, *KLF15*, *TCF4*, and *NKX2–2* have been identified as the master TFs containing both EWS-FLI1 binding motif and SE peaks [110]. Importantly, these three CRC TFs co-regulate the PI3K/AKT and MAPK signaling pathways to promote the aggressiveness of Ewing sarcoma [110].

It is worth mentioning that although SEs play an important role in stem cell identity and contribute to the development of regenerative medicine, once they are hijacked by cancer cells, their transcriptional balance is broken and the number of CSCs increases. For example, osteosarcoma-specific SEs promote tumor stemness by directly activating the expression of leukemia-inhibitory factor (LIF) [111]. In glioblastoma, the formation of a new SE-promoter loop upregulates the expression of genes associated with tumor stemness, such as *CDK6* and *SOX2* [112, 113]. In addition, SEs may also play a role in the possible response of cancer cells to chemotherapy [114, 115]. Many genes may be related to drug resistance, including characteristic genes of CSCs and some transporters, are regulated by SEs. Besides, certain factors can induce the appearance of chemoresistance by regulating the modification of histone, BRD4 and CDK activity, and the formation of SEs. Details of the mechanisms are shown in Fig. 2d.

## SE-driven mechanisms of chemoresistance

**Fig. 3:**
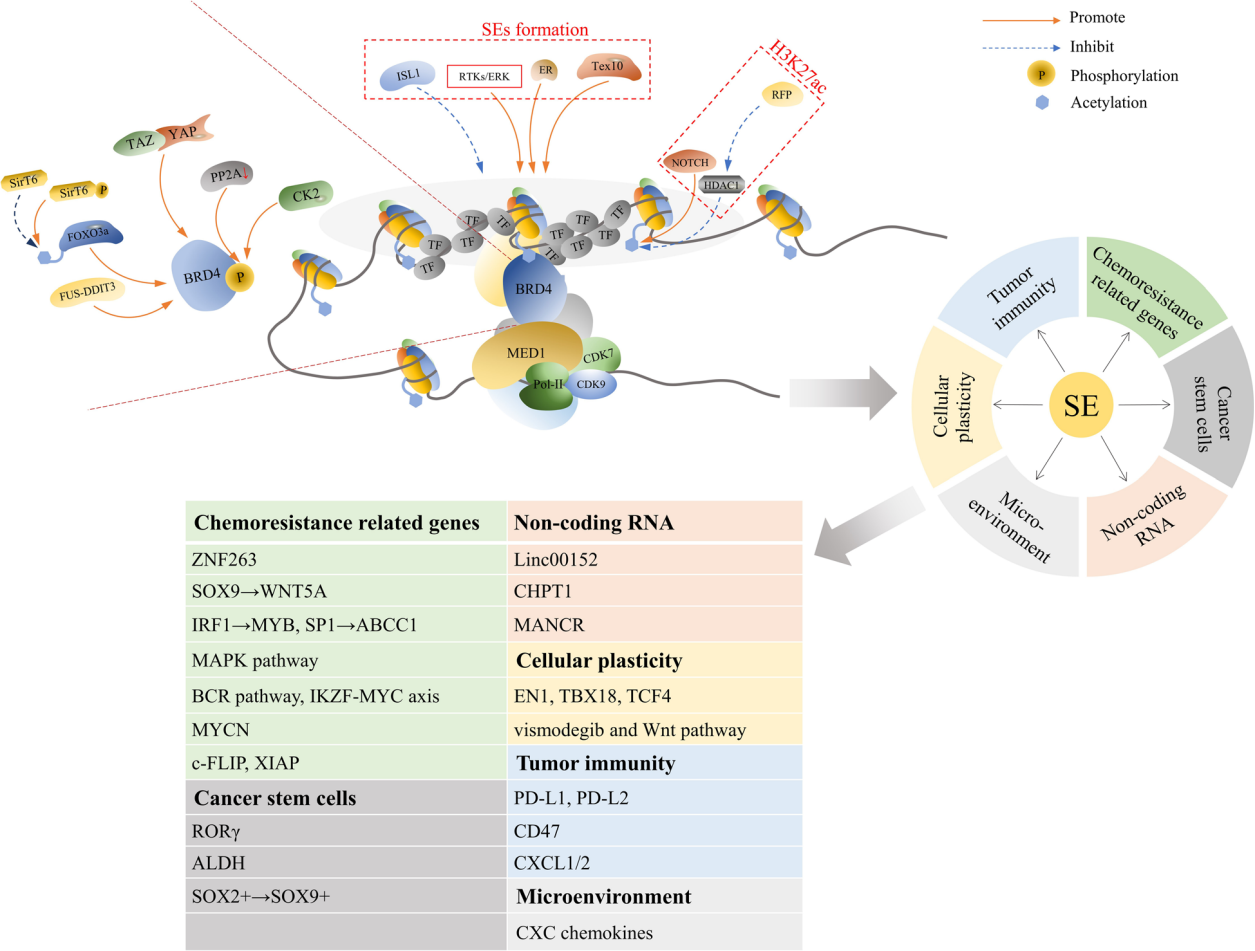
The role that SEs play in tumor chemoresistance and the factors that influence the activity of the SE complex. SEs can induce tumor chemoresistance by regulating molecular biological factors such as the formation of CSCs, cellular plasticity, the microenvironment, genes associated with chemoresistance, tumor immunity, and ncRNAs. A variety of complex components are involved in regulating the activity of SEs, including H3K27ac, BRD4, and CDKs, through which many molecules affect SE formation and activity in the process of acquiring chemoresistance. Related inhibitors can also restrain the occurrence of chemoresistance

Recent studies have shown that SEs are related to the chemoresistance of various cancers, including small-cell lung cancer (SCLC), ovarian cancer and adenocarcinoma, breast cancer, glioblastoma, and so forth [45, 116, 117]. Moreover, the sensitivity of chemotherapy can be restored by small molecule epigenetic inhibitors of SEs [45]. In the following sections, we discuss the potential association between SE-driven mechanisms and cancer chemoresistance, such as through the regulation of the formation of CSCs, cellular plasticity, the microenvironment, genes associated with chemoresistance, ncRNAs, and tumor immunity (Table 1) (Fig. 3).

**Table 1:** Functions of SEs in chemoresistance

Directions	Cancer	Resistant drugs	Induction methods	SE related genes	References
Related genes downstream	Ovarian cancer	Cisplatin	Stepwise method	SOX9 → WNT5A	[45]
	SCLC	Doxorubicin, cisplatin, etoposide	De novo	IRF1 → MYB, SP1 → ABCC1	[116]
	BRAF-mutant colon cancer	Vemurafenib	De novo	MAPK pathway	[139]
	MCL	Ibrutinib, lenalidomide	/	BCR pathway, IKZF-MYC axis	[140]
	TNBC	Neoadjuvant chemotherapy	De novo	MYCN	[141]
	NSCLC	TRAIL, cisplatin	De novo	c-FLIP, XIAP	[142]
	HCC	Sorafenib	/	ZNF263	[143]
CSCs	Pancreatic adenocarcinoma	Gemcitabine	De novo	RORγ	[117]
	Ovarian cancer	Cisplatin	Cisplatin IC20	ALDH	[119]
	Squamous cell carcinoma	Cisplatin	Cisplatin IC50	SOX2 + →SOX9+	[120]
	Breast cancer	Salinomycin	De novo	/	[121]
ncRNAs	Pan-cancer	/	De novo	Linc00152	[148]
	Prostate cancer	Enzalutamide	/	CHPT1	[149]
	Prostate cancer	/	/	MANCR	[150]
	Colorectal cancer	Oxaliplatin	2 μM oxaliplatin	MALAT1	[152]
Microenvironment	Clear cell renal cell carcinoma	/	/	CXC	[134]
	Squamous cell carcinoma	/	/	CXCL1/2	[161]
	Pancreatic ductal adenocarcinoma	/	/	/	[135]
Cellular plasticity	Breast cancer	/	/	EN1, TBX18, TCF4	[131]
	Basal cell carcinoma	Vismodegib	De novo	Vismodegib and Wnt pathway	[132]

*SCLC* small cell lung cancer, *MCL* mantle cell Lymphoma, *TNBC* triple-negative breast cancer, *NSCLC* non-small cell lung cancer, *HCC* hepatocellular carcinoma, *TRAIL* tumor necrosis factor-related apoptosis-inducing ligand

### SEs and CSCs in chemoresistance

SEs can affect the development of chemoresistance by affecting the formation and markers of CSCs. According to the literature on neuroblastoma, the SE-related genes *MEOX2*, *SIX1*, and *SIX4*, among others, are involved in CSC identity and can lead to increased resistance [21, 118]. The retinoic acid receptor-related orphan receptor gamma (RORγ) is a nuclear hormone receptor that has emerged as a key regulator of stem cells. In pancreatic adenocarcinoma, the expression of *RORγ* increases with cancer progression, and its blockade via genetic or pharmacologic approaches profoundly depletes the CSC pool and inhibits human and mouse tumor propagation by partly suppressing an SE-associated oncogenic network [117]. The high aldehyde dehydrogenase (ALDH) activity due to *ALDH1A1* expression contributes to chemotherapy resistance and tumor relapse. Studies have shown that BETi can inhibit ALDH activity by abolishing BRD4-mediated *ALDH1A1* expression through SE elements and its associated eRNAs, thereby inhibiting the growth of cisplatin-treated ovarian cancer cells [119]. For their part, SOX2 and SOX9 are stem factors that play an important role in the acquired resistance of squamous cell carcinoma (SCC) to cisplatin: the detailed mechanism of this feature is the switch from SOX2+ to SOX9+ mediated by SE remodeling [120]. A study of genome-wide RNAi screening has shown that salinomycin and JQ1 have synergy effects in the inhibition of breast CSCs, and JQ1 may be a potential small-molecule drug to overcome the resistance of cancer cells to chemotherapy [121].

### SEs and cellular plasticity in chemoresistance

Cellular plasticity refers to changes in cell genetic molecules and phenotypes, and it is a mechanism of cancer occurrence and progression [122]. The emergence of plasticity is related to the stimulation of the microenvironment, changes in cell-signaling pathways, and biochemical characteristics [123]. The most common type of cell plasticity is epithelial-mesenchymal transition (EMT) [124], which is involved in the migration, invasion, and chemotherapy resistance of cancer cells [122, 125]. Other types of plasticity, such as the transition to neuroendocrine phenotype and CSC, also appear in resistance to chemotherapy, such as SCLC and ovarian cancer [126–128].

SEs can regulate the plasticity of cancer cells and may lead to chemoresistance. The different cell subtypes of pancreatic ductal adenocarcinoma are inseparable from the modulation of SEs. Targeting SEs affects the transition from classic to basal subtypes, thereby controlling the progression of malignant tumors [129, 130]. Three TFs, namely, TBX18, EN1, and TCF4, are involved in regulating the transition in breast cancer cells from the luminal type to the basal type [131]. Resistance to Hedgehog pathway inhibitor (vismodegib) is associated with the cell identity switch of the remaining cells from the hair follicle bulge to interfollicular epidermis and isthmus mixture driven by the changing chromatin state and SE remodeling in research on basal cell carcinoma conducted by Biehs *et al*., and the simultaneous inhibition of vismodegib and the Wnt pathway can alleviate this dilemma [132].

### SEs and the microenvironment in chemoresistance

SEs are sensitive to changes in the microenvironment. A study of hair follicle stem cells found that SOX9 acts as a sensor of the microenvironment for SEs and promotes chromatin remodeling, thus providing a support for chromatin dynamics in wound repair and cell plasticity [95]. External signals in the microenvironment drive SE-related chromatin remodeling, thereby affecting cell lineage and fate, and BMP protein plays an important role in this process [133]. Likewise, SEs regulate the production of CXC chemokines in clear cell renal cell carcinoma, which mediates the release of inflammatory factors in TME and promotes inflammation and lung metastasis [134]. A study of pancreatic ductal adenocarcinoma showed that fibroblasts in the TME are upregulated by SEs, and triptolide, which acts as a CDK7 inhibitor, can downregulate SE-related genes and promote sensitivity to chemotherapy [135].

### SEs and genes associated with chemoresistance

Many genes regulated by SEs reduce the sensitivity of tumor cells to chemotherapy, either by increasing drug effluxes or by changing drug targets and other mechanisms. SOX9, a key TF related to chondrogenesis [136], hair follicles [137], and neural progenitors stemness [138], has been confirmed to be upregulated in cisplatin-resistant ovarian cancer cell lines due to the regulation of SEs, and the depletion of the regulation of SEs can lead to the downregulation of TFs associated with chemoresistance, including MAF, cMYC, ZNF430, E2F7, and KLF6, as well as the improvement of sensitivity to cisplatin [45]. Bao *et al*. identified SEs related to chemoresistance in SCLC through integrated high-throughput analyses and confirmed associated genes in resistance to doxorubicin, cisplatin, and etoposide, including *IRF1* and *SP1*, which regulate the expression of MDRPs, such as MYB and ABCC1 [116]. In BRAF-mutant colon cancer, cell resistance to vemurafenib (a BRAF inhibitor) is a result of the feedback activation for the MAPK signaling pathway by SEs, and an additional combination of related inhibitors can reverse this phenomenon [139]. In mantle cell lymphoma (MCL), SEs regulate genes related to cell survival through BRD4, such as B cell receptor (BCR) signaling and IKZF-MYC axis, and the inhibition of BRD4 may overcome MCL resistance to ibrutinib (BCR pathway inhibitor) or lenalidomide (IKZF inhibitor) [140]. MYCN, regulated by SEs, plays a key role in the tolerance of triple-negative breast cancer (TNBC) to neoadjuvant chemotherapy, where cells with high MYCN expression are more sensitive to BET inhibitors, such that the combined inhibition of BET and MEK produces a synergistic killing effect on TNBC cells [141]. The early suppression of SE-related genes, *c-FLIP*, and *XIAP*, by BET inhibitor, is effective for overcoming the resistance to tumor necrosis factor-related apoptosis-inducing ligand and cisplatin in research on non-small cell lung cancer [142]. ZNF263 is the most significant endoplasmic reticulum stress-specific SE bounding TF and has been upregulated in HCC patients and cell lines. ZNF263 knockdown results in decreased proliferation, apoptosis resistance, and chemoresistance, which implies that SE-related genes are important for chemoresistance in cancers [143].

### SEs and ncRNAs in chemoresistance

ncRNAs are important regulators in the development of the chemoresistance of various cancers, such as HCC [55, 144], colorectal cancer [56, 57], gastric cancer [58], lung cancer [59], and pancreatic cancer [60, 145]. Many studies have shown that SEs can regulate the activity of ncRNAs and change tumor progression [105, 146, 147]. SEs may trigger drug resistance through ncRNAs [102]. In a study of the role of Linc00152 in pan-cancer, it was reported that SE50407 can affect drug resistance by modulating the level of Linc00152 and then promoting AKT pathway activity [148]. CHPT1 is a tumor-promoting gene that catalyzes the synthesis of phosphatidylcholine and regulates choline metabolism. In prostate cancer cells that are resistant to enzalutamide, the enhancer element in CHPT1 SE transcribes lncRNA, namely eRNA, binds to BRD4, and regulates CHPT1 SE activity and CHPT1 expression, mediating androgen-independent drug tolerance [149]. The lncRNA MANCR has the same effect on prostate cancer, and JQ1 can downregulate MANCR to reduce cell migration and invasion [150]. Moreau *et al*. proved that hypoxia, a central mechanism in chemoresistance, can trigger oxaliplatin resistance in colorectal cancer by activating SEs and SE-derived ncRNA, MALAT1, which promotes CDH1 expression, and EMT [151, 152]. Similarly, SE-derived ncRNA, UCA1, may have anti-apoptotic effects through the Hippo/YAP1 pathway to induce chemoresistance in epithelial ovarian cancer [153].

### SEs and tumor immunity in chemoresistance

The tumor immune microenvironment (TIME) is an important contributor to the occurrence and development of cancer [154]. TIME is the main obstacle and key determinant of chemotherapy or immunotherapy checkpoint inhibitors [8], which can inhibit immune-mediated anti-tumor effects [155]. Moreover, cancer immune evasion is a major stumbling block to the design of effective anticancer immune therapeutic strategies [156]. Cancer cells can escape T-cell-mediated cellular cytotoxicity by exploiting inhibitory programmed cell-death protein 1 (PD-1)/programmed cell-death 1 ligand 1 (PD-L1) immune checkpoint [157]. Recent studies have shown that SEs play an important role in tumor immune escape and TIME.

Xu *et al*. identified a key SE (PD-L1L2-SE) located between the encoding regions for PD-L1 and PD-L2 using bioinformatic analyses and genetic manipulation. The genetic deletion of PD-L1L2-SE causes a loss of immune evasion in tumor cells and renders them sensitive to T cell killing [158]. CD47 is a cell surface molecule that inhibits phagocytosis by binding to its receptor, SIRPa, on macrophages and other immune cells [159]. Betancur *et al*. showed that cancers can evolve SEs to drive CD47 overexpression to escape immune surveillance [160]. In SCC, stem cells express and secrete CXCL1 and CXCL2 by establishing SEs, which send a signal to the immune system to consolidate cell stability in the TIME [161]. Inhibition of related SEs may increase the sensitivity of cancer cells to immunotherapy or overcome chemoresistance by changing the TIME.

## Regulation of SEs complex activity to overcome chemoresistance of cancer

The phenomenon of cancer chemoresistance and low mutation frequency demonstrates the importance of epigenetic modification. Increasing evidence implies that chemotherapy can induce SE-driven transcriptional programs to maintain the chemoresistant state [18, 45]. Therefore, targeted inhibitors that specifically block the interaction between SE regions and their corresponding complexes can rescue upregulated oncogene- and chemoresistance-related genes [162, 163].

In relation to the different protein components in the regulatory pathway, SE inhibitors are divided into multiple types: BRD4 inhibitors, histone acetylation inhibitors, CDK inhibitors, and gene-editing technology [20, 62]. Because the first three are mostly small-molecule inhibitors that can effectively prevent the interaction of SEs and the complex and have greater feasibility, they are more widely used [164]. Furthermore, the extensive effects of master TFs, histone modification, and cofactors make it difficult to target them, while mediator complexes such as CDK7 and BRD4 are relatively characteristic [62]. Here, we summarized the chemoresistant mechanisms involved with SEs, as well as the effects of the SE-related inhibitors on reverse drug resistance and combined sensitization (Tables 2 and 3).

**Table 2:** Regulation of SE activity in chemoresistance

Complex	Cancer	Resistant drugs	Induction methods	SE-associated genes	Mechanisms	References
H3K27ac	Glioblastoma	Temozolomide	50 μM temozolomide	/	Transient resistant state	[183]
	Glioblastoma	Temozolomide	/	RFP/HDAC1	Inhibit H3K27ac	[184]
	Leukemia	/	/	Notch	Promote H3K27ac	[186, 187]
BRD4	Breast cancer	AKTi	Stepwise method	SirT6, FOXO3a	BRD4/FOXO3a/CDK6 axis	[170]
	Melanoma	Vemurafenib	1 μM vemurafenib	YAP/TAZ	Transcription addition mediated by YAP/TAZ through BRD4	[171]
	Myeloma	IMiDs	/	PP2A	Hyper pBRD4	[172]
	TNBC	BETi	Stepwise method	CK2, PP2A	pBRD4 increase MED1 recruitment	[173]
	T cell leukemia	GSI	1 μM GSI	NDME→BDME	Transition from NDME to BDME	[174]
	Liposarcoma	Trabectedin	de novo	FUS-DDIT3	Formation of CRC	[175]
	PDAC	5-FU	Stepwise method	HMGA2	/	[176]
	MCL	Ibrutinib, venetoclax and palbociclib	De novo	E3-ubiquitin ligase	/	[177]
CDK	B cell lymphoma	ABT-199	20 nM ABT-199	BCL2 18q21 loss	Drug-tolerant “persister” state	[191]
	Leukemia cells	BETi	/	RNA pol-II, MYC	/	[193]
	Anaplastic thyroid carcinoma	Doxorubicin	De novo	DNA damage repair	Downregulation of DNA damage repair	[192]
SEs formation	TNBC	Trametinib	/	RTKs/ERK	SEs de novo formation	[194]
	Hepatocellular carcinoma	Sorafenib, cisplatin	5 μM/L sorafenib/cisplatin	Tex10	Formation of ESC related SEs	[195]
	ER+ breast cancer	Endocrine therapy	Doxycycline	ER-ligand-independent	Increased combination of ER and SEs	[196]
	ER+ breast cancer	Endocrine therapy	Endocrine therapy	Endogenous cholesterol biosynthesis	Epigenetic reprogramming	[197]

*TNBC* triple-negative breast cancer, *PDAC* pancreatic ductal adenocarcinoma, *MCL* mantle cell lymphoma, *AKTi* AKT inhibitor, *IMiDs* immunomodulatory drugs, *BETi* BET bromodomain inhibitors, *GSI* gamma-secretase inhibitor, *NDME* notch-dependent MYC enhancer, *BDME* BRD4-dependent MYC enhancer, *RNA pol-II* RNA polymerase-II, *TSA* trichostatin, *ESC* embryonic stem cell, *CRC* core transcription regulatory circuitry

**Table 3:** Reversal of chemoresistance

Target	Inhibitors	Cancers	Resistant drugs or sensitized drugs	References
BRD4	JQ1	Ovarian cancer	Cisplatin	[45, 119]
		NSCLC	TRAIL, cisplatin	[142]
		Breast cancer	Salinomycin	[121]
		Breast cancer	AKTi	[170]
		Melanoma	Vemurafenib	[171]
		PDAC	5-FU	[176]
	I-BET151	MCL	Ibrutinib, lenalidomide	[140]
		TNBC	Trametinib	[194]
	I-BET762	NSCLC	TRAIL, cisplatin	[142]
	OTX-015	NSCLC	TRAIL, cisplatin	[142]
	SR2211	Pancreatic adenocarcinoma	Gemcitabine	[117]
	ARV-771	MCL	Ibrutinib, venetoclax, palbociclib	[177]
	MS417	Breast cancer	AKTi	[170]
H3K27ac	TSA	Glioblastoma	Temozolomide	[183]
CDK7	THZ1	B cell lymphoma	ABT-199	[191]
CDK12	THZ531	Anaplastic thyroid carcinoma	Doxorubicin	[192]

*NSCLC* non-small cell lung cancer, *PDAC* pancreatic ductal adenocarcinoma, *MCL* mantle cell lymphoma, *TNBC* triple-negative breast cancer, *AKTi* AKT inhibitor, *TRAIL* tumor necrosis factor-related apoptosis-inducing ligand

### BRD4 inhibitors

Bromodomain and extra terminal (BET) protein family, including BRD1, BRD2, BRD3, BRD4, and BRDt, can recognize histone proteins by binding to acetylated lysine residues and play a role of reader in epigenetic regulation [165, 166]. Among these, BRD4 is an important element in cancer biology and can interact with SEs. A colocalization of BRD4 and MED1 appears at H3 acetylation chromatin sites, particularly H3K27 [166], so the factors that influencing BRD4 and MED1 also affect the transcriptional activity regulated by SEs.

BRD4 inhibitors can be divided into JQ1 and its derivatives, 3,5-dimethylisoxazole derivatives, 2-thiazolidinone derivatives, and others, based on their chemical structure [167]. JQ1 is the first bromodomain and extra-terminal domain (BET) inhibitor (BETi) that competitively inhibits the binding of BRD4 to chromatin, leading to cell cycle arrest and increased apoptosis [167, 168]. Many *in vivo* and *in vitro* studies have shown that JQ1 is effective against a variety of cancers [169]. In this review, we found that BETi can reverse drug resistance and has a synergistic effect with some chemotherapy drugs.

AKT inhibitor (AKTi) is a class of drugs targeted to breast cancer, but, its frequent use could disturb the regulatory mechanisms of common tumor cells and induce drug resistance [170]. As has been found in related research, AKTi treatment increases the acetylation of FOXO3a by dephosphorylating SirT6 and induces the combination of FOXO3a and BRD4. This series of reactions increases the transcription of CDK6, which promotes the development of drug resistance to AKTi [170]. The BRD4 inhibitors JQ1 and MS417 improve the growth-suppressive effect mediated by AKTi, and the BRD4/FOXO3a/CDK6 axis passivates AKT inhibition in luminal breast cancer [170]. The resistance of melanoma cells to BRAF inhibitor (vemurafenib) is associated with transcription addiction, and the mechanism of this resistance is that YAP/TAZ induces the recruitment of SEs to BRD4 and RNA Pol-II and activates the expression of growth-regulating genes [171]. In myeloma cells sensitive to immunomodulatory drugs (IMiDs), the depletion of IKZF1/IKZF3 caused SE instability and reduced the binding of BRD4. However, in resistant cells, the binding of BRD4 to SEs was unaffected, which could be attributed to the decrease in PP2A activity and the increase in BRD4 phosphorylation [172]. In addition, the phosphorylation of BRD4 is also related to BETi tolerance in TNBC cells, the mechanisms for which include a decrease in PP2A activity and an increase in CK2 activity and MED1 recruitment [173]. In T cell leukemia, Notch1 could activate the expression of downstream genes by binding to SEs of MYC. However, in cell lines that are resistant to a gamma-secretase inhibitor, the inhibition of Notch1 cannot cause the downregulation of MYC. A study by Yashiro-Ohtani *et al*. indicated that this is due to the transition of MYC SEs from Notch1-dependent (NDME) to BRD4-dependent MYC enhancer [174] (Fig. 3).

Studies have shown that FUS-DDIT3 has regulatory effects on SEs through interaction with BRD4, which may participate in the resistance of liposarcoma cells to trabectedin and CRC formation, and BET inhibitors can effectively overcome this limitation in treatment [175]. Furthermore, the blockage of BRD4 sensitizes 5-FU toxicity to pancreatic ductal adenocarcinoma [176]. ARV-771, a proteolysis-targeting chimera of BET protein has stronger activity in interfering with BET protein than BETi, which may be promising for the overcoming of the resistance of MCL cells to ibrutinib, venetoclax, and palbociclib [177].

### Histone acetylation

Chemical modifications of DNA and histone proteins in chromatin could modulate gene expression through changing conformations and altering transcriptional complex recruitment. Common chemical methods of modifying histone proteins include acetylation/deacetylation and methylation/demethylation [166]. Acetylated histones destabilize nucleosomes, thereby increasing the accessibility of chromatin to TFs [178, 179]. Acetylation modification of chromatin histones is jointly regulated by histone acetyltransferase and histone deacetylase (HDAC) enzymes; the two are in a state of dynamic balance [180]. High-density H3K27ac is a sign that identifies SEs, which leads to a rapid response of target genes to various signals [21, 181, 182].

Studies have shown that resistance is associated with histone acetylation. Rabé *et al*. confirmed that the transient resistance state to temozolomide in glioblastoma cells is related to high levels of histone acetylation and chromatin remodeling, and the sensitive and resistant state shows lower acetylated histone levels. The combined application of temozolomide and HDAC inhibitor trichostatin could prevent the transition from a transient to a resistant state [183]. Similarly, a review of the resistance mechanism of glioblastoma cells to temozolomide indicated that disrupting the formation of RFP/HDAC1 complex would interfere with the function of cis-regulatory-element, controlled by H3K27ac, and then it would overcome the chemoresistance induced by SE-related genes [184, 185]. In leukemia and T-cell acute lymphoblastic leukemia, the tolerance of chemotherapy by cancer cells is related to the regulation of Notch1 protein to H3K27 acetylation. The mutation of *Notch1* would suppress H3K27ac marks on SEs and disrupt downstream MYC expression, which may show how resistant cells maintain growth under drug pressure [186, 187] (Fig. 3).

### CDKs

The appearance of SEs in cancer cells leads to high transcription output and high transcription addiction, which result in stronger responses to transcriptional inhibition [188]. CDKs are an important category of protein, which can bind to cyclin proteins and regulate the cell cycle, playing an important role in gene transcription [189]. These features make them indispensable for the regulation of SEs activity and overcoming chemoresistance. Studies have shown that SEs activate transcriptions are inseparable from the recruitment of CDK7-containing TFIIH (a transcription initiation complex), CDK9-containing p-TEFb (a transcription extension complex), and CDK12 [86, 97, 190]. Therefore, inhibitors that target CDKs can reduce SE activity, thereby inhibiting the occurrence and progression of cancer and reversing chemoresistance.

According to research on B cell lymphoma, the emergence of a drug-tolerant “persister” state is associated with SE remodeling in resistance to ABT-199, a target drug of BCL-2, and the inhibitor of CDK7 (THZ1) could significantly reverse this effect [191]. One study showed that the CDK12 inhibitor THZ531 can inhibit transcriptional extension and downregulate DNA damage repair, thereby increasing the sensitivity of anaplastic thyroid carcinoma cells to doxorubicin [192]. The combination of the BETi inhibitor and the CDK7 inhibitor leads to the synthetic lethality in leukemia cells resistant to BETi, which is associated with the RNA pol-II activity regulated by SEs [193] (Fig. 3).

### Other links to SE activity

In the case of drug resistance, the appearance and regulation of SEs are also affected by other factors. As a response to trametinib, a MEK1/2 inhibitor, adaptive resistance takes place in TNBC as a result of de novo SE formation [194]. Tex10 upregulates ESC-related SEs in sorafenib- and cisplatin-resistant cell lines, which is an important chemoresistance mechanism for HCC [195]. Studies have shown that the ER-ligand-binding domain is mutated in ER^+^ breast cancer cells that are resistant to endocrine therapy, and these cells acquire ligand-independent growth. During the exploration of the mechanism of this phenomenon, it was found that the interaction between ER and SEs in the mutant cells has increased [196]. Furthermore, epigenetic reprogramming for endocrine therapy activates endogenous cholesterol biosynthesis, which promotes the constitutive activation of ERα in drug-resistant cells [197].

Moreover, inhibitors of oncogenes can both directly influence the expression of oncogenes and block genome-wide oncogene enhancers and SEs activation as well, along with downstream transcriptional signaling. A recent study found that darolutamide, an inhibitor of the androgen receptor, antagonizes androgen signaling by blocking enhancers and SE activation in prostate cancer [198]. The new drugs involved in SE-related oncogenes’ transcriptional regulation may produce important results for chemotherapeutic resistance. The remodeling of SEs in drug-resistant cells may also be related to the downregulation of certain genes. In ovarian cancer, ISL1, a lineage determinant, is downregulated when cells are continuously stimulated by cisplatin, mediating the increase in CSCs and chemoresistance induced by SE plasticity [199] (Fig. 3).

## Perspective and summary

Due to the differing genetic/epigenetic backgrounds and heterogeneity of tumors, the efficacy of chemotherapy varies widely across patients. Understanding the changing epigenetic landscape during chemotherapy and the dynamic interaction between the genetic and epigenetic machinery in response to chemotherapy are inevitable for assessing the clinical efficacy of chemotherapy. Within the new frontier of epigenetic modifiers, more and more evidence has shown the important role of SEs in tumor development and chemotherapy resistance.

Epigenetic gene signatures, particularly SEs, have attracted increased interest lately with regard to the molecular subtypes of tumors and their prediction of tumor recurrence, the prognosis of tumor patients, and chemotherapy resistance in different cancers. Mapping the epigenome and monitoring epi-biomarkers (such as SEs) using genome-wide analyses at clinical settings before, during, and after treatment and at relapse will help evaluate and adjust the treatment approach and design personalized epigenetic therapy [11]. Despite the continuous emergence of relevant research, chemotherapy resistance remains a complex process that needs to be explored in depth. We may still need to conduct more research upstream and investigate more initial mechanisms to clarify the reasons for the generation and regulation of resistance-related SEs. Furthermore, related inhibitors require clinical trials to prove their effectiveness and safety for overcoming chemoresistance.

## Conclusions

In conclusion, SEs are central to the maintenance of identity of cancer cells and promote SE-driven-oncogenic transcriptions to which cancer cells become highly addicted. Chemotherapeutics induce SEs reprogramming in cancer cells, converting a transient transcriptional state into a stably resistant one. Aberrant transcriptional regulation of SEs plays important roles in epigenetic mechanisms of cancer chemoresistance via the formation of CSCs, cellular plasticity, the microenvironment, genes associated with chemoresistance, ncRNAs, and tumor immunity. This dependence on SE-driven transcription to maintain chemoresistance offers an Achilles’ heel for chemoresistance. Indeed, the inhibition of SE components dampens oncogenic transcription and inhibits tumor growth to ultimately achieve combined sensitization and reverse the effects of drug resistance. The research on the SEs in tumorigenesis and chemoresistance may help find new drugs to overcome chemoresistance from the bench to the bedside.

## Data Availability

Available.

## Abbreviations

TME: Tumor microenvironment
CSC: Cancer stem cells
MDRP: Multi-drug resistant protein
SE: Super-enhancer
TF: Transcription factor
ESC: Embryonic stem cell
ncRNA: non-coding RNA
tRNA: transfer RNA
lncRNA: long non-coding RNA
eRNA: enhancer RNA
seRNA: super-enhancer RNA
RNA pol-II: RNA polymerase-II
H3K27ac: Histone H3 lysine27 acetylation
CDK7: Cyclin-dependent kinase 7
BRD4: Bromodomain-containing protein 4
H3K4me1: Histone H3 lysine4 methylation
CRC: Core transcriptional regulatory circuitry
SD: SE domain
TAD: Topologically associating domain
CTCF: CCCTC-binding factor
IDR: Intrinsically disordered region
MED1: Mediator of RNA Pol-II transcription subunit 1
TCF4: Transcription factor 4
RORγ: Receptor-related orphan receptor gamma
ALDH: Aldehyde dehydrogenase
EMT: Epithelial-mesenchymal transition
SCLC: Small-cell lung cancer
MCL: Mantle cell lymphoma
BCR: B cell receptor
TNBC: Triple-negative breast cancer
HCC: Hepatocellular carcinoma
TIME: Tumor immune microenvironment
PD-1: Programmed cell-death protein 1
PD-L1: Programmed cell death 1 ligand 1
SCC: Squamous cell carcinoma
BET: BRD and extraterminal domain
BETi: BET protein inhibitor
AKTi: AKT inhibitor
IMiDs: Immunomodulatory drugs
HDAC: Histone deacetylase
